# Food web differences between two neighboring tropical high mountain lakes and the influence of introducing a new top predator

**DOI:** 10.1371/journal.pone.0287066

**Published:** 2023-06-13

**Authors:** José Luis Jiménez-Seinos, Javier Alcocer, Dolors Planas

**Affiliations:** 1 Programa de Posgrado en Ciencias del Mar y Limnología, Universidad Nacional Autónoma de México, Ciudad de México, México; 2 Grupo de Investigación en Limnología Tropical, FES Iztacala, Universidad Nacional Autónoma de México, Ciudad de México, México; 3 Centre de Recherche en Géochimie et Géodynamique GEOTOP, Montreal, Canada; University of Eldoret, KENYA

## Abstract

High mountain lakes (HMLs) are considered unique and comparable ecosystems for monitoring global climate change. The food web structure can indicate the response of these ecosystems to ecological threats, such as fish introduction, by analyzing the trophic dynamics. Nonetheless, the food webs of tropical HMLs are less well-studied than temperate HMLs. The present study assessed the food webs of two neighboring (600 m apart) tropical HMLs, El Sol and La Luna, inside the crater of the Nevado de Toluca volcano, Mexico. It used stable isotopes (δ^13^C and δ^15^N) and Bayesian mixing models with different trophic discrimination factors and priors to assess the impacts of introduced rainbow trout, persisting only in the larger lake, El Sol. The food web in Lake El Sol was more complex than in Lake La Luna, mainly due to its larger size, extensive vegetated littoral zone, and being fueled by autochthonous primary production. In contrast, the smaller and fishless Lake La Luna has a reduced and bare littoral zone that harbored a simple food web substantially sustained by allochthonous carbon inputs. The persistence of introduced rainbow trout in Lake El Sol but not in Lake La Luna accentuated the differences between the lakes. The models suggested that rainbow trout fed on key consumers of littoral macroinvertebrates (70–80%) and pelagic zooplankton (20–30%), increasing the linkage between sub-networks. In both tropical HMLs, the species richness and herbivorous fraction were elevated compared with temperate HMLs, while the linkage density and omnivorous fraction were lower. Basal nodes dominated these tropical HMLs, and the vegetated littoral zone of Lake El Sol had more intermediate (omnivore) nodes. Our results showed the convenience of food web analysis to compare the effects of introduced fish in originally fishless lakes in different latitudes.

## Introduction

High mountain lakes (HMLs) are the highest water bodies in the world, located above the tree line, at more than 3000–4000 m a.s.l. at tropical and subtropical latitudes [1, 2]. The high altitude results in unique and harsh environmental features such as a high incidence of solar and UV radiation, oligotrophy, isolation, and marked habitat heterogeneity, leading to different patterns in local diversity among HMLs within the same district and the adaptations of organisms to altitude [1–3].

Although HMLs are generally remote, human activities also threaten these environments through air-borne pollution from local and long-range transport, pollution within their drainage basins, climate change, and the stocking of exotic fish in otherwise fishless lakes [2]. Fish stocking strongly influences food web patterns of inland water bodies, including HMLs, and is an important threat to, for example, endemic species [4]. Effects on food webs differ according to local conditions: food web topology may be simplified by a reduction in the diversity of top predators and their consumption of local species [4, 5] or may be made more complex by the addition of new trophic links if the introduced fish is an omnivore [6].

An introduced fish species could increase trophic interactions between littoral and pelagic food webs in a lake with a vegetated littoral shore by directly consuming benthic organisms [7, 8]. Concurrently, macrophytes and macroalgae provide habitat for primary and secondary producers, food for benthic invertebrates, and shelter for zooplankton [9, 10], promoting a foraging habitat for fish, especially in lakes with extended and gently sloped littoral zones [11]. The reliance of fish on zoobenthos is higher in small lakes where the littoral zone is the dominant habitat [8, 12].

The introduction of rainbow trout into natural fishless lakes creates a "cascade effect" in the pelagic food web by consuming herbivorous zooplankton, which reduces grazing pressure on algae and increases populations of inedible phytoplankton [4, 13]. Moreover, fish stocking adversely affects the ecological characteristics of herbivorous prey such as *Daphnia* (e.g., their size, phenology, morphology) [12, 14].

Analysis of food webs in HML ecosystems has used gut contents of consumers, meta-analyses of published literature, and, rarely, stable isotope analysis (e.g., [15–17]), but little of this information concerns tropical environments. More recently, analysis of tracer isotopes (δ^13^C and δ^15^N) and mixing models have been widely used to identify diet sources of consumers in natural environments [17–19], as well as autochthonous or allochthonous supplies [20], isotopic fractionation [21, 22], food chain length, and trophic position [23].

Mixing models based on a probabilistic Bayesian framework provide general explanatory information about the proportional contribution of different sources to a mixture. These models analyze the source and trophic discrimination factor (TDF) variability, the population structure of the consumer, and error assumptions [24–27]. Mixing models are sensitive to variation in TDF. Therefore, an incorrect TDF can lead to erroneous results in mixing models and a misunderstanding of trophic relationships between consumers and sources [28].

This study used a methodology that combines stable isotopes and ecological data to construct food webs. We aimed to understand the littoral and pelagic food web structure of two adjacent oligotrophic tropical HMLs with a similar origin, geology, oxygen concentration, and temperature but different community structure and the influence of fish introduction. Through visual prey selection, we hypothesize that rainbow trout introduction alters pelagic and littoral food webs in Lake El Sol (from now on, El Sol). In addition, the trout feed alternately on littoral and pelagic food sources and promote coupling between pelagic and littoral zones. In contrast, the homogeneous steep and naked littoral zone in Lake La Luna (after this La Luna) should limit the abundance and diversity of food sources, reflected in a simple food web with few nodes and trophic interactions and a shortened food chain.

## Materials and methods

### Study site

**Fig 1:**
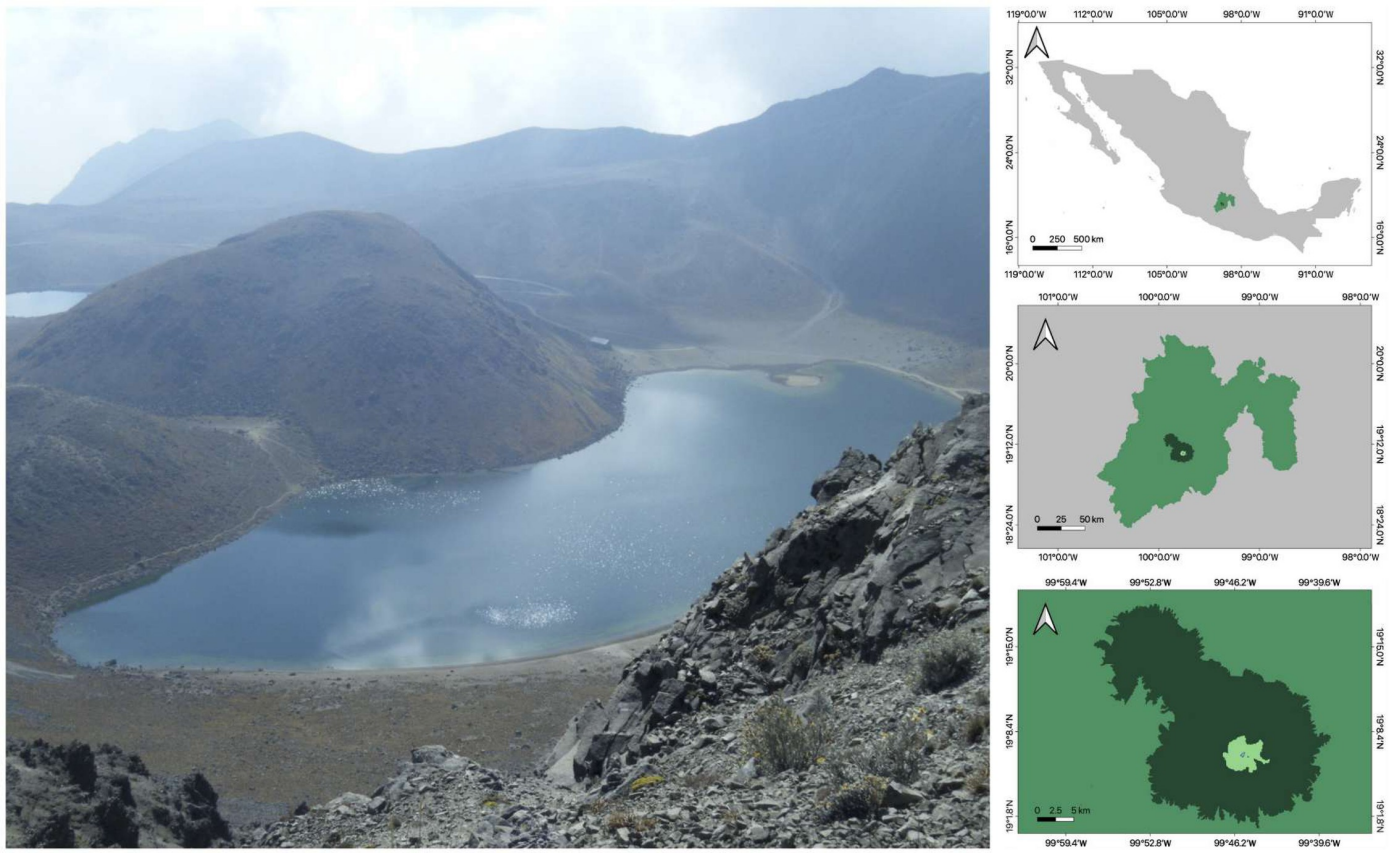
Study site. Tropical high-mountain Lakes El Sol (front) and La Luna (back) inside the Nevado de Toluca volcano crater, Central Mexico. (Map data set obtained from open access topographic and toponymic vector data set, scale 1:100,000, National Institute of Statistics and Geography, Mexico at https://www.inegi.org.mx/app/mapas/).

El Sol and La Luna are tropical HMLs within the Nevado de Toluca volcano crater (19°06’ N, 99°45’ W) at 4200 m a.s.l. (Fig 1). The Nevado de Toluca is the fourth-highest volcano in Mexico within the Trans-Mexican Volcanic Belt, the principal and highest volcanic mountain range in Mexico [29]. It has an alpine climate (Ew) [30, 31]. Despite their proximity (600 m) and the common origin and climate, the two lakes differ in morphometry, water chemistry [32], thermal regime, and, markedly, in their ecological communities [33–36]. In the 1950s, the Mexican government introduced rainbow trout (*Oncorhynchus mykiss)* into both lakes, but the species persisted only in El Sol [37]. Both lakes have marked differences in morphometry; El Sol is larger (surface: 19 ha versus 3 ha) and deeper (maximum depth: 12 m versus 10 m) with an extensive shallow, heterogeneous littoral zone, including vegetated and rock-dominated areas. La Luna has a small homogeneous steep non-vegetated littoral zone (bucket-shaped) with a small shore development. Both lakes are warm polymictic, discontinuous in El Sol and continuous in La Luna [38], and have no winter ice cover. Physicochemical and morphometric variables are summarized in (S1 Table).

### Sampling

We used vertical hauls to collect phytoplankton (20 μm mesh) and zooplankton (70 μm mesh). Samples were sieved through 100 μm mesh to remove large zooplankton and retain filamentous algae. We removed the small zooplankton and macroalgae (≤100 μm) from the samples with a fine pipette and stereomicroscope. Isolated phytoplankton samples were then collected on pre-combusted GF/A glass fiber filters [39, 40]. Littoral benthic macroinvertebrates (BMIs) were collected with an acrylic corer (2.4 x 2.5 cm) at the vegetated and bare littoral zones and sieved through a 500 μm mesh. Using a stereomicroscope, we separated the dominant zooplankton and BMI species with a 500 μm pipette and entomology forceps. A sample of total zooplankton was also included in determining the isotopic concentration of bulk zooplankton (>70 μm). We maintained zooplankton and BMIs in pre-filtered lake water for 2 hours to allow gut evacuation [15, 40]. We collected macrophytes and macro-algae by hand, stored them in whirl-pack sample bags, and rinsed them three times with pre-filtered water. Epiphytes colonized macrophytes; then, we considered the macrophyte-epiphytes complex in the analysis. Rainbow trout were caught with a fishing rod, stored on the ice at 4°C, and frozen in the laboratory after sampling. For isotope analysis, we removed the transverse white dorsal muscle sample without skin or bones [41].

### Statistical and stable isotope analysis

To calculate the isotope concentration (δ^13^C and δ^15^N) and the C:N ratio, all biological samples were frozen at -20°C, freeze-dried, and ground to a fine powder with an agate mortar and pestle. Samples were then analyzed in an IRMS (Isotopic Ratio Mass Spectrometer Isoprime 100TM, CF-EA) at the GEOTOP laboratory (Montreal, Canada) using two international references, *VPDB* for carbon and *AIR* for nitrogen. For δ^13^C isotopes, two internal reference materials (δ^13^C = -28.73±0.06‰ & -11.85±0.04‰) were used to normalize the results on the NBS19-LSVEC scale. A third reference material (δ^13^C = -17,04±0,11‰) was analyzed as an unknown to assess the exactness of the normalization. Results are given in delta units (δ) in ‰ vs. VPDB. The overall analytical uncertainty (1s) is better than ±0.1‰. For nitrogen isotopes (δ^15^N), two internal reference materials (δ^15^N = -0.10±0.24‰ & +14.95±0.09‰) were used to normalize the results on the AIR (IAEA-N1, IAEA-N2 & IAEA-N3) scale. A third reference material (δ^15^N = -0,1±0,15‰) was analyzed as an unknown to assess the exactness of the normalization. Results are given in delta units (δ) in ‰ vs. AIR. The overall analytical uncertainty (1s) is better than ±0.2‰.

We compared the isotopic values of the two lakes by the Mann-Whitney U test since the Shapiro-Wilk test showed that the δ^13^C of El Sol and the δ^15^N of La Luna had a non-normal distribution. The skewed δ^15^N distribution in La Luna was caused solely by the extremely low value of the dominant Zygnemataceae macroalga *Temnogametum iztacalense*. We also performed a dissimilarity test (ANOSIM) to compare the isotopic values of both lakes and zones (littoral and pelagic), considering δ^13^C and δ^15^N values. We analyzed the relationship between rainbow trout (adults: 33.48 ± 2.7 cm) weight (independent variable) and isotope values (dependent variable) with linear regression models.

We used a two-source food web equation to calculate the food chain length using the δ^15^N values of top consumers and baselines [23]. In El Sol, we used rainbow trout as the top consumer, the copepod *Leptodiaptomus cuauhtemoci* in the pelagic zone, and the gastropod *Physa* sp. for the littoral as a baseline. In La Luna, we used the cladoceran *Daphnia ambigua* as the top consumer and phytoplankton as the baseline. We calculated the trophic level of primary consumers from their average trophic discrimination factor (TDF) and the average δ^15^N value of their sources as baseline [23]. For the stocked lake, we used the average difference between δ^13^C and δ^15^N values of rainbow trout and its potential food sources to calculate the TDF [18, 22, 42]. We estimated the relationship between food sources and TDF with linear regressions, using δ^13^C and δ^15^N values of diet sources as independent variables and carbon and nitrogen TDFs as dependent variables [21].

### Bayesian model construction for introduced rainbow trout diet

**Fig 2:**
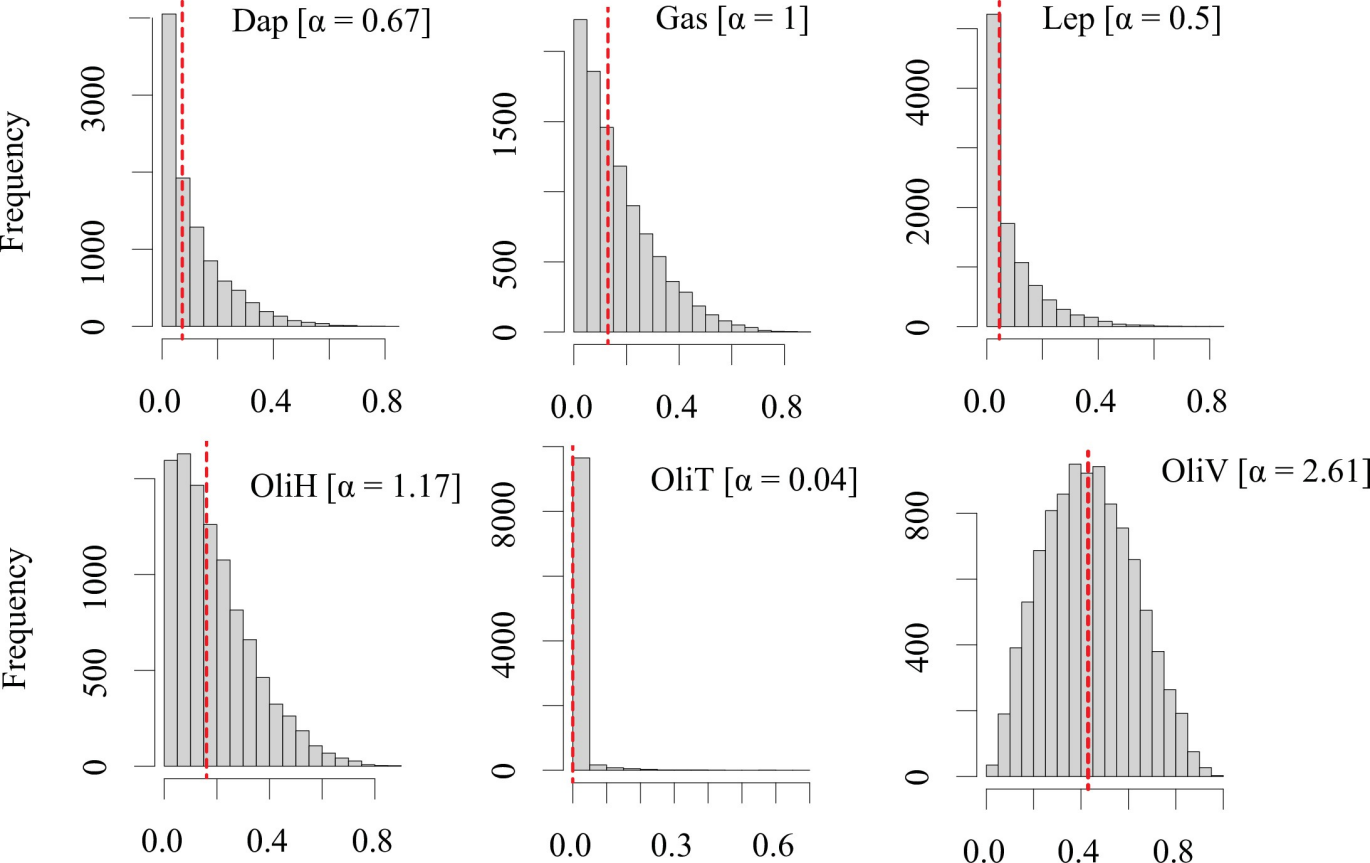
Biomass prior to Dirichlet distribution. Standardized Dirichlet distribution of macroinvertebrates. Source biomass was used as an informative prior distribution of the six possible rainbow trout diet sources within the littoral zone of Lake El Sol. Redlines show the median value of the distribution. (Dap = *Daphnia ambigua*, Gas = *Phys* sp., Lep = *Leptodiaptomus cuauhtemoci*, OliH = *Limnodrilus hoffmeisteri*, OliT = *Tubifex tubifex*, OliV = *Lumbriculus variegatus*).

We constructed Bayesian mixing models (BMMs) with the MixSIAR R package using δ^13^C and δ^15^N as isotopic tracers to estimate the proportional contribution of potential food sources to the trout diet [25, 43]. We assumed a random effect for the consumer and did not account for population structure (differences in feeding behavior due to hierarchical organization, sex, or size) because all fish collected were adults capable of moving around the lake to feed. In addition, a multiplicative error was used to account for sampling bias and differences inherent in the predation process [27]. To measure the effect of TDF on food source contribution and model accuracy, we used four different published TDFs and one calculated TDF for δ^13^C and δ^15^N [15, 44–46]. We used two different priors: the first or generalist prior considered the same probability for fish to feed on all sources and was constructed with a uniform Dirichlet distribution using a hyperparameter alpha (α = 1) and divided by the number of sources (∑α) [25]; the second or biomass prior considered a differing probability of food sources to be eaten depending on their biomass within El Sol. We standardized the average annual biomass in mg C m^- 2^. For zooplankton biomass, we took the reported average biomass [35] and multiplied it by the zooplankton carbon percentage measured in this study. For benthic organisms, we used the reported biomass directly [47], except for gastropods, because this is the first study that sampled and reported gastropods (*Physa* sp.) in El Sol; for those, we used an average biomass value to calculate a neutral hyperparameter α and to avoid further bias in their contribution to the rainbow trout diet. Biomass values were standardized by the total number of sources (∑α) divided by the total biomass to build a less aggressive prior distribution and maintain the same mean (Fig 2) [25, 48].

We calculated statistical significance with the Gelman-Rubin convergence test; this analyzes Markov Chain Monte Carlo (MCMC) tested by the Bayesian method (JAGS) until the value is <1.1 and MCMC chains converge or where all desired posterior distributions have been already tested and are closer to 1. We also estimated convergence with the Gelweke test, which compared the means between the first (10%) and second (50%) parts of MCMC chains. We assumed convergence if their means were significantly similar [24, 25]. We used the widely applicable information criterion (WAIC) and leave-one-out (LOO) cross-validation to test out-of-sample prediction accuracy [49]. Finally, we validated the Bayesian mixing models using the in-polygon assumption, which was developed to determine whether a proposed mixing model is logical and includes all consumers within a Bayesian simulation of mixing polygons constructed with the isotopic values of food sources [50].

### Community structure

We analyzed the community structure of each lake, using functional groups and trophic guilds to assign each taxon into ecological clusters at different trophic levels [51, 52]. We based a literature search on global databases and previous studies of the two lakes on proposing potential trophic interactions for fish, zooplankton, primary producers, and benthic macroinvertebrate taxa (S2 Table). We also added trophic links using Bayesian mixing models (rainbow trout) and stable isotope similarity between analyzed groups and species (primary consumers and detritivores). We assigned the trophic level of each node using the calculated food chain length and trophic level [23].

We calculated the food web topology and metrics with the Cheddar R package [43, 53] using ecologic groups as nodes. We proposed trophic links within a n x n matrix with predators as columns and prey as rows, where 1 means a trophic interaction and 0 a lack of it [6]. Finally, we used food web metrics to compare El Sol and La Luna lakes with each other and with temperate HMLs [6].

### Ethics statement

The National Protected Area (APFF Nevado de Toluca), CONANP, gave informed, written consent by signing a permit for conducting scientific research on the lakes.

## Results

### Stable isotope analyses and trophic structure

**Fig 3:**
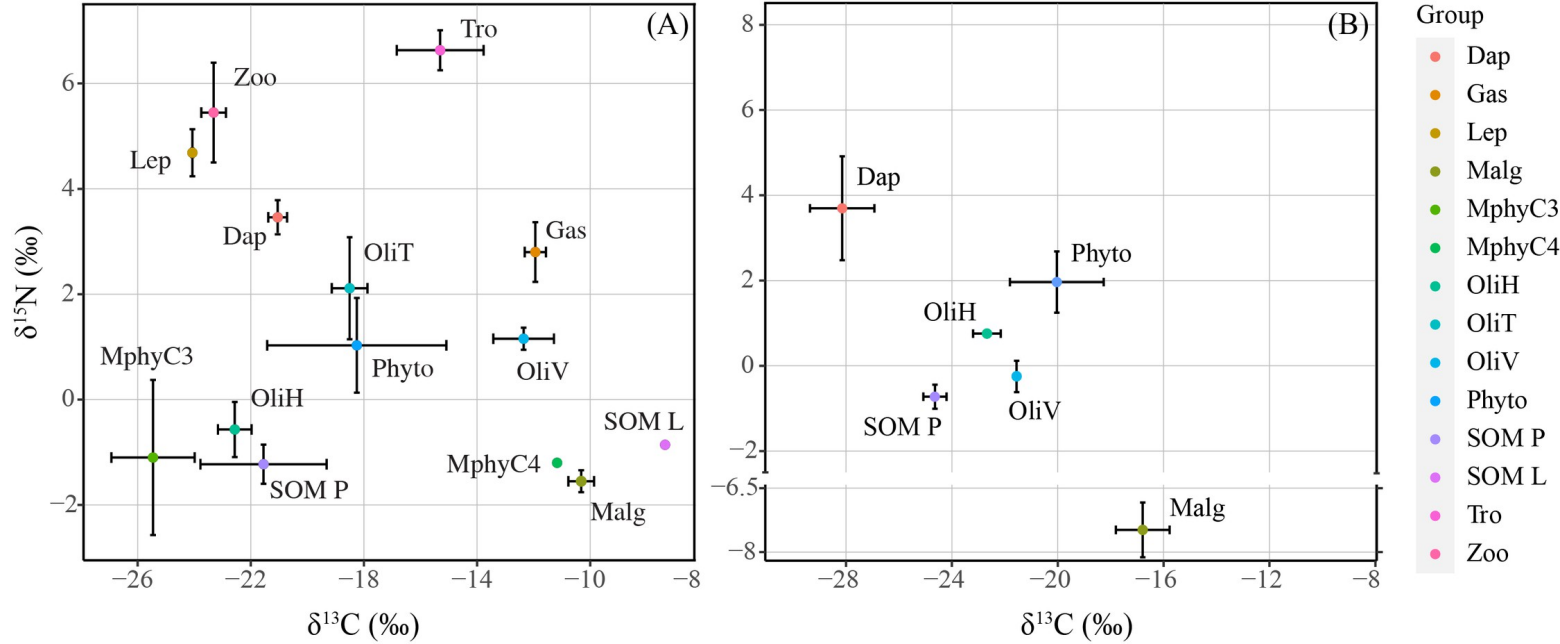
Stable isotope values of the major Lakes El Sol and La Luna groups. δ^13^C and δ^15^N values (mean and standard deviation) of single species and aggregated groups (zooplankton, phytoplankton) in Lakes El Sol (A) and La Luna (B). Dap = *Daphnia ambigua*, Gas = *Physa* sp., Lep = *Leptodiaptomus cuauhtemoci*, Malg = macroalgae (Oedogoniaceae and Zygnemataceae), MphyC_3_ = macrophytes with C_3_ metabolism, MphyC_4_ = macrophytes with C_4_ metabolism, Phyto = phytoplankton, SOM = sediment organic matter (L = littoral and P = profundal), OliH = *Limnodrilus hoffmeisteri*, OliT = *Tubifex tubifex*, OliV = *Lumbriculus variegatus*, Tro = *Oncorhynchus mykiss*, Zoo = bulk zooplankton.

El Sol had a more complex food web, where benthic consumers had higher δ^13^C values than pelagic ones (W = 125, p<0.001), and δ^15^N values were higher for pelagic consumers (W = 0, p<0.001), rainbow trout was in the middle of both zones, indicating a coupling role between them (Fig 3A). Differences between the δ^13^C values of the two oligochaetes in fish stocked El Sol indicated different food sources: *L*. *hoffmeisteri* (-22.57 ± 0.59‰) was associated with sediment organic matter (-21.55 ± 2.23‰) and *L*. *variegatus* (-12.35 ± 1.07‰) was associated with macrophytes with C4 metabolism (-11.17‰) and filamentous macroalgae (-10.32 ± 0.3‰) (Fig 3A). Among the pelagic components in El Sol, δ^13^C values of phytoplankton (-18.25 ± 3.17‰) and *D*. *ambigua* (-21.05 ± 0.1‰) showed a higher similarity compared with *L*. *cuauhtemoci* (-24.1 ± 3.17‰), suggesting that *D*. *ambigua* is the principal phytoplankton consumer in El Sol. The δ^13^C value of *Physa* sp. (-11.94 ± 0.14‰) was similar to those of C4-metabolism macrophytes (-11.17‰) and filamentous macroalgae (-10.32 ± 0.3‰), suggesting a littoral herbivorous feeding behavior in El Sol (S2 Table).

Just six major groups represented La Luna. We considered SOM profundal because the littoral zone was not well delimited, and pelagic consumers had higher δ^15^N values than benthic. In comparison, benthic consumers had higher δ^13^C values than pelagic ones (Fig 3B). δ^13^C values were different by 1.12‰ between the two oligochaetes sampled in La Luna; *Limnodrilus hoffmeisteri* had δ^13^C values similar to those of SOM (△δ^13^C = 1.97‰), suggesting a detritivorous diet as reported in the literature (S2 Table) and *Lumbriculus variegatus* had values similar to those of phytoplankton (△δ^13^C = 1.52‰).

We obtained 14 trophic groups in El Sol and six in La Luna from stable isotope analysis (S3 Table); common groups in both lakes were bulk phytoplankton, cladocerans (*Daphnia ambigua*), BMI (*Lumbriculus variegatus* and *Limnodrilus hoffmeisteri*), benthic macroalgae and sedimentary organic matter (Fig 3). Isotopic δ^13^C values of littoral and pelagic common groups were different in both lakes (W = 119, p<0.001), and components in the fish-stoked El Sol were enriched compared to the values of the fishless La Luna (Table 1). δ^15^N had no significant difference between both lakes’ common groups (W = 310, p = 0.7). When comparing the two isotope tracers (δ^13^C and δ^15^N), ANOSIM showed differences between both lakes, indicating that isotopic values were more similar within each lake than between them (R = 0.18, p = 0.0017). The isotopic values of major comparable groups (Fig 3), considering both lakes, were also significantly different, independent of the lake (R = 0.56, p< 0.001), but no significant differences were found between zones (pelagic and littoral) (R = 0.03, p = 0.29). Phytoplankton δ^13^C values in El Sol were 7.78% higher than the phytoplankton δ^13^C values of La Luna.

**Table 1:** Stable isotope values of groups and species in two high mountain, tropical Lakes El Sol and La Luna (Mean ± standard deviation).

Lake	Group/species	δ^13^C	δ^15^N	Lake	Group/species	δ^13^C	δ^15^N
El Sol	*Oncorhynchus mykiss*	-15.3 ± 1.54	6.63 ± 0.37	El Sol	Bulk phytoplankton	-18.25 ± 3.17	1.03 ± 1
El Sol	*Lumbriculus variegatus*	-12.35 ± 1.07	1.16 ± 0.21	El Sol	*Limnodrilus hoffmeisteri*	-22.6 ± 0.6	-0.57 ± 0.52
El Sol	*Tubifex tubifex*	-18.6 ± 0.63	2.11 ± 1	El Sol	Profundal sedimentary organic matter	-21.6 ± 2.23	-1.2 ± 0.37
El Sol	*Physa* sp.	-11.94 ± 0.14	2.8 ± 0.57	El Sol	Littoral sedimentary organic matter	-7.35	-0.9
El Sol	*Daphnia ambigua*	-21.05 ± 0.1	3.46 ± 0.2	La Luna	*Daphnia ambigua*	-28.15 ± 1.22	3.7 ± 1.22
El Sol	*Leptodiaptomus cuauhtemoci*	-24 ± 0.035	4.7 ± 0.45	La Luna	*Lumbriculus variegatus*	-21.6 ± 0.035	-0.25 ± 0.21
El Sol	Bulk zooplankton	-23.32 ± 0.44	5.45 ± 0.95	La Luna	*Limnodrilus hoffmeisteri*	-22.7 ± 0.27	0.8 ± 0.034
El Sol	Macroalgae	-10.32 ± 0.3	-1.55 ± 0.21	La Luna	Macroalgae	-16.8 ± 1.01	-7.5 ± 0.64
El Sol	Macrophytes C_3_	-25.46 ± 1.47	-1.1 ± 1.5	La Luna	Profundal sedimentary organic matter	-24.7 ± 0.2	-0.73 ± 0.13
El Sol	Macrophytes C_4_	-11.17	-1.2	La Luna	Bulk phytoplankton	-20.04 ± 1.78	1.96 ± 0.72

### Rainbow trout trophic discrimination factor (TDF)

The calculated TDF for rainbow trout white muscle, considering pelagic and littoral potential sources, was 0.49 ± 4.7‰ for δ^13^C and 4.43 ± 1.32‰ for δ^15^N. The difference in δ^13^C values between rainbow trout tissue and littoral sources was smaller (-1.6‰ ± 2.8‰) than for pelagic sources (7.25 ± 2.12‰). In rainbow trout tissues, ^13^C values were higher than in its diet sources except for *Physa* sp. and the oligochaete *L*. *variegatus*; consequently, both sources were loaded with a negative carbon TDF for the Bayesian models. As expected, δ^15^N values of rainbow trout (6.63 ± 0.37‰) were higher than in all potential food sources. In contrast, the difference in δ^15^N values between rainbow trout tissue and littoral sources (5 ± 0.85‰) was higher than for pelagic sources (2.6 ± 0.76‰). The littoral sources had higher δ^13^C values and then closer to rainbow trout δ^13^C values than pelagic sources, which in turn had higher δ^15^N values and closer to rainbow trout δ^15^N values.

### Rainbow trout diet—Bayesian multivariate meta-analysis

**Fig 4:**
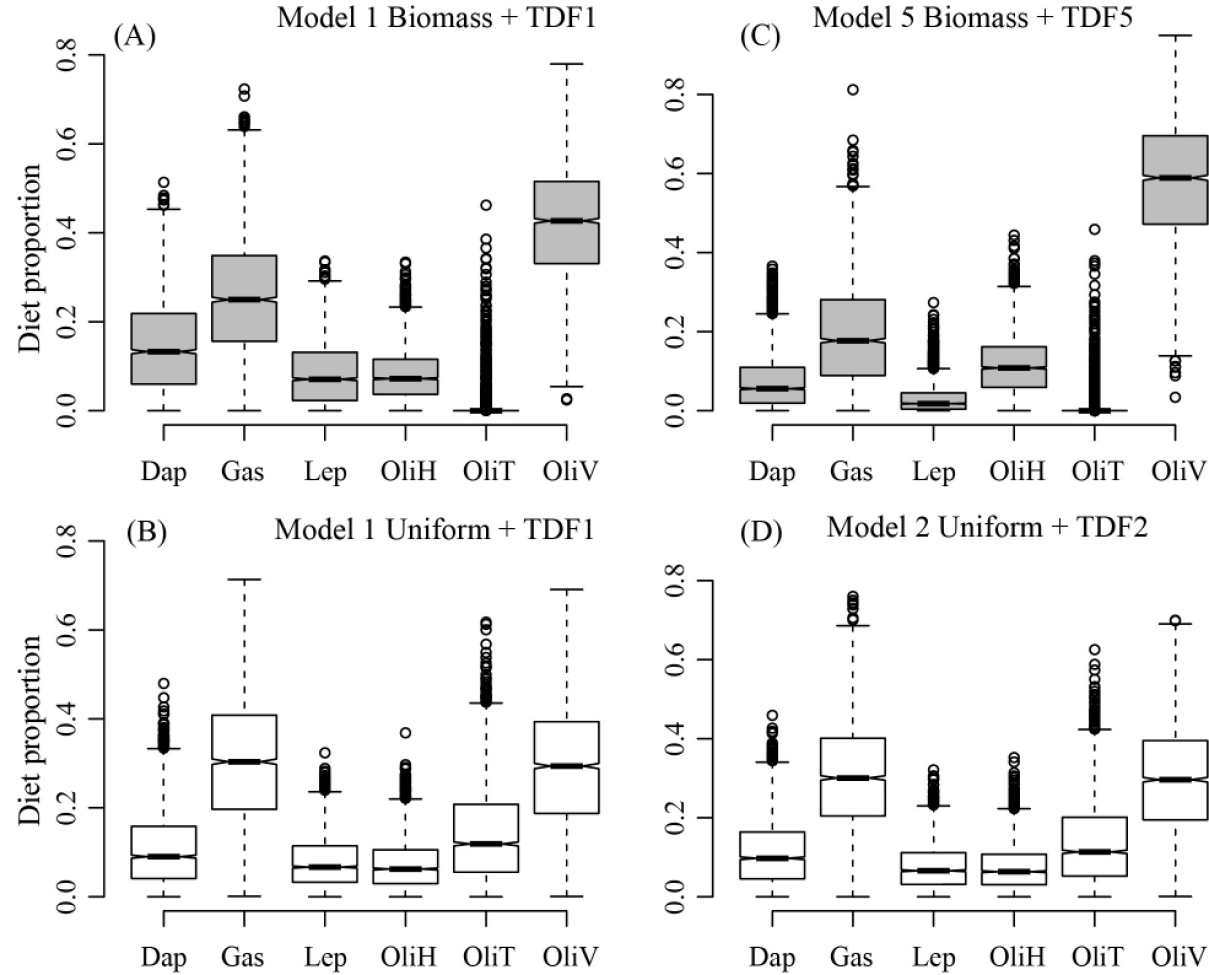
The proportional contribution of six food sources to rainbow trout diet. The proportional contribution of food sources to rainbow trout diet in El Sol. (A) Model 1 using TDF1 = (△δ^13^C = 0.49 ± 4.7‰, △δ^15^N = 4.43 ± 1.32‰) and biomass prior, (B) TDF1 = (△δ^13^C = 0.49 ± 4.7‰, △δ^15^N = 4.43 ± 1.32‰) and uniform prior, (C) Model 5, TDF5 = (△δ^13^C = 2.8 ± 1.5‰, △δ^15^N = 5.14 ± 1.35‰) and biomass prior, (D) Model 2, TDF2 = (△δ^13^C = 0.8 ± 1.1‰, △δ^15^N = 3 ± 2.6‰) and uniform prior.

Statistical tests performed after multivariate meta-analysis showed a convergence among MCMC chains for all models. All variables were < 0.01 in Gelman-Rubin and Gelweke tests, and the three chains were < 6 and closer to 1, showing a significant similarity between the means of tested MCMC chains. The better models explaining the isotope data values of rainbow trout diet using Leave-one-out comparison and meeting Bayesian mixing polygon assumption were Model 1 with biomass and uniform priors, Model 5 with biomass prior, and Model 2 with a uniform prior (Fig 4A–4D).

According to Model 1, oligochaetes were the main prey for rainbow trout in El Sol using calculated TDF and biomass prior, with *L*. *variegatus* as the most important diet source (0.42 ± 0.13). In comparison, *Tubifex tubifex* (0.01 ± 0.035) and *L*. *hoffmeisteri* (0.81 ± 0.056) made a lower contribution (Fig 4A). Gastropods (*Physa* sp.) were the second most significant diet source (0.26 ± 0.14); these grazers, with a slightly higher δ^15^N and trophic position than detritivores (*L*. *hoffmeisteri*) or detritivore-herbivores (*L*. *variegatus*), would be a significant food source for rainbow trout. The biomass of gastropods is unknown, and their contribution is more uncertain than other sources. However, it was essential to incorporate the group in the analysis because of their high abundance in the vegetated littoral zone during the sampling. Cladocerans and copepods accounted for 0.023 of the adult rainbow trout diet. The main zooplanktonic sources were *D*. *ambigua* (0.15 ± 0.1) and *L*. *cuauhtemoci* (0.08 ± 0.07) (Fig 4A). For all models, the MIB *L*. *variegatus* and *Physa* sp. had a higher contribution to the rainbow trout diet, and *D*. *ambigua* had a higher contribution than *L*. *cuauhtemoci*, considering pelagic sources (Fig 4A–4D). The contribution of *T*. *tubifex* was higher in models with uniform priors, while *L*. *hoffmeisteri* contributed with a higher proportion in models using a biomass prior (Fig 4A–4D).

### Food web structure

The littoral sub-network harbored the major differences in food web topology between the two lakes. Littoral links were higher in the stocked lake with an extended littoral vegetated zone (El Sol littoral 50, El Sol pelagic 37), and the number of links in both sub-networks, number of nodes, links, and species richness of BMI was higher in El Sol (Table 2). Pelagic links were higher than littoral links in the small fishless lake (La Luna pelagic 23, La Luna littoral 15). Littoral and pelagic sub-networks had more links between them in El Sol than in La Luna, revealing a higher coupling in the fish-stocked lake (El Sol 11, La Luna 6). Secondary consumer nodes were present only in El Sol (rainbow trout and *Hydra vulgaris*). In contrast, herbivorous nodes were present in both lakes and slightly higher within the pelagic zone, representing the higher fraction and contributing to the most significant number of links (Table 2).

**Table 2:** Food web parameters based on the theoretical analysis of Lakes El Sol and La Luna.

Parameter	Notation	El Sol	La Luna	Parameter	Notation	El Sol	La Luna
Species richness	**(S)**	261	128	Fish nodes	**(N**_**N**_ **)**	1	0
Nodes	**(N)**	42	31	Herbivorous fraction		0.38	0.41
Links	**(L)**	99	56	Omnivorous fraction		0.15	0.06
Linkage density 1	**(D = L/S)**	0.38	0.44	Primary producers’ fraction		0.35	0.39
Linkage density 2	**(D = L/N)**	2.36	1.75	Predators fraction		0.14	0.03
Potential links	**(Lp = N** ^**2**^ **)**	1764	961	Phytoplankton richness	**(S**_**P**_ **)**	197	70
Directed connectance	**(C = L**/N^2^)	0.06	0.04	Zooplankton richness	**(S**_**Z**_ **)**	41	32
Basal nodes	**(N**_**P**_ **)**	15	13	Benthic macroinvertebrate richness	**(S**_**I**_ **)**	22	4
Zooplankton nodes	**(N**_**Z**_ **)**	10	10	Fish richness	**(S**_**N**_ **)**	1	0
MIB nodes	**(N**_**I**_ **)**	12	5	Trophic levels		3	2

## Discussion

Despite their proximity and similar origin, El Sol and La Luna have marked food web differences at all trophic levels. A larger size, rainbow trout stocking, and an extensive vegetated littoral zone in the food web of the originally fishless tropical HML El Sol mirrored the results reported for other lakes, including HMLs in cold and temperate regions [4, 6] where active foraging by introduced fish has increased food web coupling. We observed a higher reliance of rainbow trout on littoral BMI, but zooplankton was also an important food source. The latter was evident in (Fig 3A) where rainbow trout and its diet sources draw an A-frame shape with pelagic sources on the left and benthic on the right considering top consumers as the top of the shape (Fig 3A), as observed in other omnivorous predators coupling pelagic and benthic pathways [54].

The food chain length in El Sol was longer (2.86), and its food web network was more complex, with higher taxonomic richness, number of links, nodes, and trophic levels (Table 2). The complexity was associated with the heterogeneous littoral zone of El Sol, which harbored a higher abundance and diversity of BMI. Littoral complexity affects invertebrate diversity and depends on the fish’s presence and food preferences [55, 56]. Littoral complexity can decrease zoo-planktivorous fish abundance by reducing the zooplankton (mainly filter feeders) food sources and increasing algae and periphyton, which compete with phytoplankton [55]. The effect of littoral complexity differs for zoobenthic and omnivorous fish, such as rainbow trout in El Sol, which use more littoral food sources when the habitat is more complex (higher presence of macrophytes) and zooplankton abundance is lower [54, 56]. The increase in BMI functional feeding groups is related to microhabitat complexity; BMI density increases with complexity but just with fish presence [56]. Both effects (fish presence and littoral complexity) could be the main drivers of a higher number of nodes and links in the littoral zone of El Sol. However, the few BMI predators can be associated with fish presence since their density positively relates to littoral complexity in lakes without fish [56].

Intermediate macrophyte cover can enhance the diversity and density of BMI compared with low vegetation or non-vegetated areas [56, 57]. The absence of macrophytes in La Luna (sparse filaments of *Temnogametum iztacalense*) could be associated with a lower density and diversity of BMI. Also, BMI with significative biomass were detritivores, and no path of autochthonous macroalgae as a food source was observed. The marked differences in the isotopic ratios, the short food chain (1.9), and the simple food web structure of La Luna suggest that the absence of omnivorous and secondary consumers and the low linkage between pelagic and benthic sub-networks intensify a separation in the trophic interactions and isotope values of its food web components, similar to the food web characteristics reported for ultra-oligotrophic lakes, acid lakes, and non-vegetated reduced littoral lakes [39, 58, 59].

The models indicated that the direct contribution of zooplankton to adult trout in El Sol ranges from 0.2 to 0.3 in diet proportion (Fig 4). Differently, the gut contents analysis of trout introduced into temperate Italian HMLs [13] showed a higher diet proportion (0.39). The present results show the importance of adult fish as consumers of the zooplankton population, as in other temperate mountain lakes [13]. Introduced fish can change the size structure of zooplankton from large-bodied cladocerans and copepods to smaller ones and rotifer-dominated communities through size-preferential consumption in temperate lakes [4, 13, 60] and HMLs [13, 61]. In the fish-stocked lake (El Sol), the most abundant zooplankton species, *D*. *ambigua* and *L*. *cuauhtemoci* were similar in size (948 ± 156 μm). On the contrary, in the fishless lake (La Luna), the cladoceran *D*. *ambigua* was notoriously larger (1,648 ± 277 μm) and pigmented, differing from *D*. *ambigua* in El Sol, which was hyaline. We suggest that this difference between lakes reflects the presence or absence of fish, as observed in other temperate lakes [5, 62].

The significantly lower δ^13^C of comparable major groups in La Luna was most likely associated with higher terrestrial inputs (allochthonous) from the surrounding catchment. Allochthonous inputs as a food source are frequent in unproductive lakes [63, 64]. In addition, the dominance of mixotrophic algae in the pelagic zone [36] and the dissimilarity between *D*. *ambigua* and phytoplankton δ^13^C values supported the importance of allochthonous organic matter as a carbon source in the food web of La Luna. In contrast, in El Sol, the δ^13^C values of herbivorous zooplankton (e.g., *D*. *ambigua*) and phytoplankton were similar, and food web components of all trophic levels were enriched in C^13^. This pattern in isotopic signals is probably associated with zooplankton reliance on autochthonous primary producers, higher primary production, and trophic status [40, 65].

The enriched phytoplankton δ^13^C values in El Sol suggest that epiphytic algae and unattached macroalgal filaments from the littoral zone (Oedogoniaceae and Zygnemataceae algae) were incorporated into the pelagic zone as suggested by previous studies. Cuna et al. (2022) [36] found filaments of littoral algae (Oedogoniaceae and Zygnemataceae) in El Sol’s plankton. Also, 87.5% of Bacillariophyceae morphospecies were tychoplanktonic.

### Food web topology of tropical HMLs El Sol and La Luna versus temperate HMLs

Tropical HMLs El Sol and La Luna showed species richness at all trophic levels and functional groups that differed from temperate HMLs [6]. The species richness of phytoplankton and zooplankton was higher in the tropical HMLs, while benthic macroinvertebrates were more diverse in temperate ones. The omnivorous fraction was the most important in temperate HMLs and was associated with food web stability [6, 66]. With an extended littoral zone and introduced fish, the larger lake (El Sol) had a higher omnivorous fraction than the smaller fishless lake (La Luna) (Table 3). Omnivore nodes were associated with the littoral zone; hence, rainbow trout may decrease food web stability, as observed in temperate lakes [67] and HMLs [68]. Nevertheless, macrophyte beds in the stocked lake El Sol represented an important basal resource and habitat that would increase the littoral sub-web complexity by allowing more trophic interactions [59, 69].

**Table 3:** Comparing tropical Lakes El Sol and La Luna food web metrics with temperate HMLs [6]. (S = species richness, L = number of links, C = connectance, D = linkage density, Omn = omnivores fraction, Herb = herbivores fraction and B = basal species fraction, Lat = latitude, Tro = tropical, Tem = temperate, Fish = presence/absence of fish, Om = *Oncorhynchus mykiss*, Sf = *Salvelinus fontinalis*, St = *Salmo trutta*), No = fishless).

Lake	(S)	(L)	(C)	(D)	Omn	Herb	B	Lat	Fish
**El Sol, Mexico**	261	99	0.06	2.36	0.15	0.4	0.36	Trop	Om
**La Luna, Mexico**	128	56	0.06	1.75	0.06	0.41	0.39	Trop	No
**Caballeros, Spain**	64	344	0.08	5.4	0.84	0.18	0.34	Temp	No
**Cimera, Spain**	85	645	0.09	7.6	0.91	0.06	0.16	Temp	Sf
**Grande de Gredos, Spain**	96	534	0.06	5.6	0.93	0.05	0.52	Temp	St

The herbivorous fraction was the most important in El Sol and La Luna. Among temperate HMLs, the herbivorous fraction was higher in the fishless lake Grande de Gredos (Table 3). Connectance has been reported as low for temperate and tropical HMLs, being equal in both tropical lakes and the temperate lake Grande de Gredos with native fish, while slightly higher in Caballeros (fishless) and Cimera (introduced trout) (Table 3). Considering that higher species richness and low connectance potentially decrease the vulnerability of food webs to disturbances [70], El Sol would be potentially more resistant to disturbance than La Luna, and both tropical HMLs would be potentially more resistant than their temperate counterparts. On the contrary, considering that lower vulnerability is also associated with a higher number of intermediate species [70], temperate HMLs are potentially less vulnerable, having an elevated number of omnivorous intermediate species, compared with the two tropical lakes (El Sol and La Luna) where herbivorous and basal species were high (Table 3).

### Rainbow trout diet model and TDF comparison

We calculated a higher δ^15^NTDF (4.43 ± 1.32‰) and lower δ^13^CTDF (0.49 ± 4.7) than those previously published for rainbow trout [41, 71], but closer to the generally used δ^13^CTDF, 0.8 ± 1.1‰ [45]. These results, suggest that omnivorous fish like trout in tropical HMLs with a diet based mainly on benthic macroinvertebrates could have a higher TDF than commonly stated.

To improve accuracy, recent Bayesian mixing models of diet using stable isotope tracers have frequently included priors based on aspects of ecological data such as behavior, traditional knowledge of feeding ecology, stomach contents, or literature on food web topology [6, 17, 72]. The only way to find better prior assumptions and avoid those priors that introduce more uncertainty to the analysis is by comparison of Bayesian models with informative and uniform priors [73]. The fully Bayesian information criterion (leave-one-out validation) showed a higher weight (Loo_w_) for Model 1 using the biomass prior and TDF calculated in this study (Table 4), corroborating the use of accurate priors [49]. Models 1, 4, and 5 had a higher weight for models calculated with the biomass prior, but models 2 and 3 had a higher weight for models calculated with a uniform prior (Table 4). The in-polygon Monte Carlo simulation [50] showed that all models except Model 4 (using the TDF reported by [41]) were accurate for rainbow trout diet estimation, where all sources were within the mixing polygon 95% area (S1 Fig).

**Table 4:** Comparison of rainbow trout diet models using the leave-one-out method, considering six possible sources with uniform or biomass prior assumptions, and using different TDFs. OliV: *Lumbriculus variegatus*, OliT: *Tubifex tubifex*, OliH: *Limnodrilus hoffmeisteri*, Gas: *Physa* sp., Dap: *Daphnia ambigua*, Lep: *Leptodiaptomus cuauhtemoci*. TDF1 = (△δ^13^C = 0.49 ± 4.7‰, △δ^15^N = 4.43 ± 1.32‰), TDF2 = (△δ^13^C = 0.8 ± 1.1‰, △δ^15^N = 3 ± 2.6‰) [44, 45], TDF3 = (△δ^13^C = 0.91 ± 1.04‰, △δ^15^N = 3.41 ± 0.2‰) [46], TDF4 = (△δ^13^C = 1.85‰, △δ^15^N = 2.54‰) [41], TDF5 = (△δ^13^C = 2.8 ± 1.5‰, △δ^15^N = 5.14 ± 1.35‰) [15]. LOOic = Leave-one-out information criterion, se_LOOic = standard error of leave-one-out information criterion, dLOOic = difference leave one-out value compared with the best-fitted model, se_dLOOic = difference in the standard deviation of the leave-one-out value compared with the best-fitted model.

Model ID	Model sets	LOOic	se_LOOic	dLOOic	se_dLOOic	weight
Mod 1	Biomass + TDF 1	3.3	3	0	NA	0.144
Mod1 Null	Uniform + TDF1	3.5	3.1	0.2	0.6	0.13
Mod 4	Biomass + TDF2	3.5	3.6	0.2	0.8	0.13
Mod 5	Biomass + TDF5	3.5	3.3	0.2	1.1	0.13
Mod 2 Null	Uniform + TDF2	3.7	3.1	0.4	0.6	0.118
Mod 2	Biomass + TDF2	4.2	3.1	0.9	0.2	0.092
Mod 3 Null	Uniform + TDF3	4.5	3.9	1.2	1.4	0.079
Mod 3	Biomass + TDF3	5	3.5	1.7	1.3	0.061
Mod 4 Null	Uniform + TDF4	5	3.8	1.7	1.6	0.061
Mod 5 Null	Uniform + TDF5	5.2	3.5	1.9	1.8	0.056

The results highlight the importance of estimating a correct TDF because the outcome of a mixing model depends on the mean and variance of TDF. Thus, accurate predictions on diet contributions to the consumer must be weighted on model design and cross-validation [61, 66]. The similar patterns in the contribution of possible sources to fish diet observed in this study, even with different model assumptions [74], strongly suggest that the use of different TDF values is a reliable alternative to Bayesian mixing methods in the analysis of fish diets in a tropical HML if isotopic data and fractionation studies are scarce.

## Conclusions

The food web topology of the larger, vegetated, and fish-stocked El Sol was more complex than the smaller, non-vegetated, and fishless La Luna. Both the pelagic and littoral benthic food webs of El Sol were strongly linked with the C4 photosynthetic path. Fueled mainly by autochthonous primary production, El Sol exhibited a complex food web primarily associated with an extensive vegetated littoral zone. Differently, the smaller lake (La Luna), with a reduced and bare littoral zone, showed a simple food web and was linked mainly with allochthonous carbon paths.

The rainbow trout diet comprised predominantly (up to 0.8) littoral benthic macroinvertebrates and was complemented with herbivorous zooplankton in the pelagic zone. Rainbow trout introduction fostered the differences between the lakes by lengthening the food chain in El Sol and linking the pelagic and the benthic zones. At the same time, connectance and linkage density remained similar.

There were differences in the food webs between tropical HMLs El Sol and La Luna and temperate HMLs, with higher species richness and lower linkage density in the former. Also, the omnivorous fraction was markedly lower, and the herbivorous fraction was higher in tropical El Sol and La Luna than in other temperate HMLs.

## Supporting information

S1 Fig Mixing polygons calculated with Bayesian analysis for diet sources of rainbow trout. (TIFF)

S1 Table Physicochemical, trophic, and morphometric variables of Lakes El Sol and La Luna. (DOCX)

S2 Table Ecologic groups used for the food web construction of Lakes El Sol and La Luna. (DOCX)

S3 Table Minimal data set of stable isotope data (δ^13^C and δ^15^N) for the major Lakes El Sol and La Luna groups. (DOCX)
