# Supplementary figures and images for: Food web differences between two neighboring tropical high mountain lakes and the influence of introducing a new top predator
Source: PLoS One. 2023 Jun 13;18(6):e0287066. doi: 10.1371/journal.pone.0287066 (PMC10263323; doi:10.1371/journal.pone.0287066)

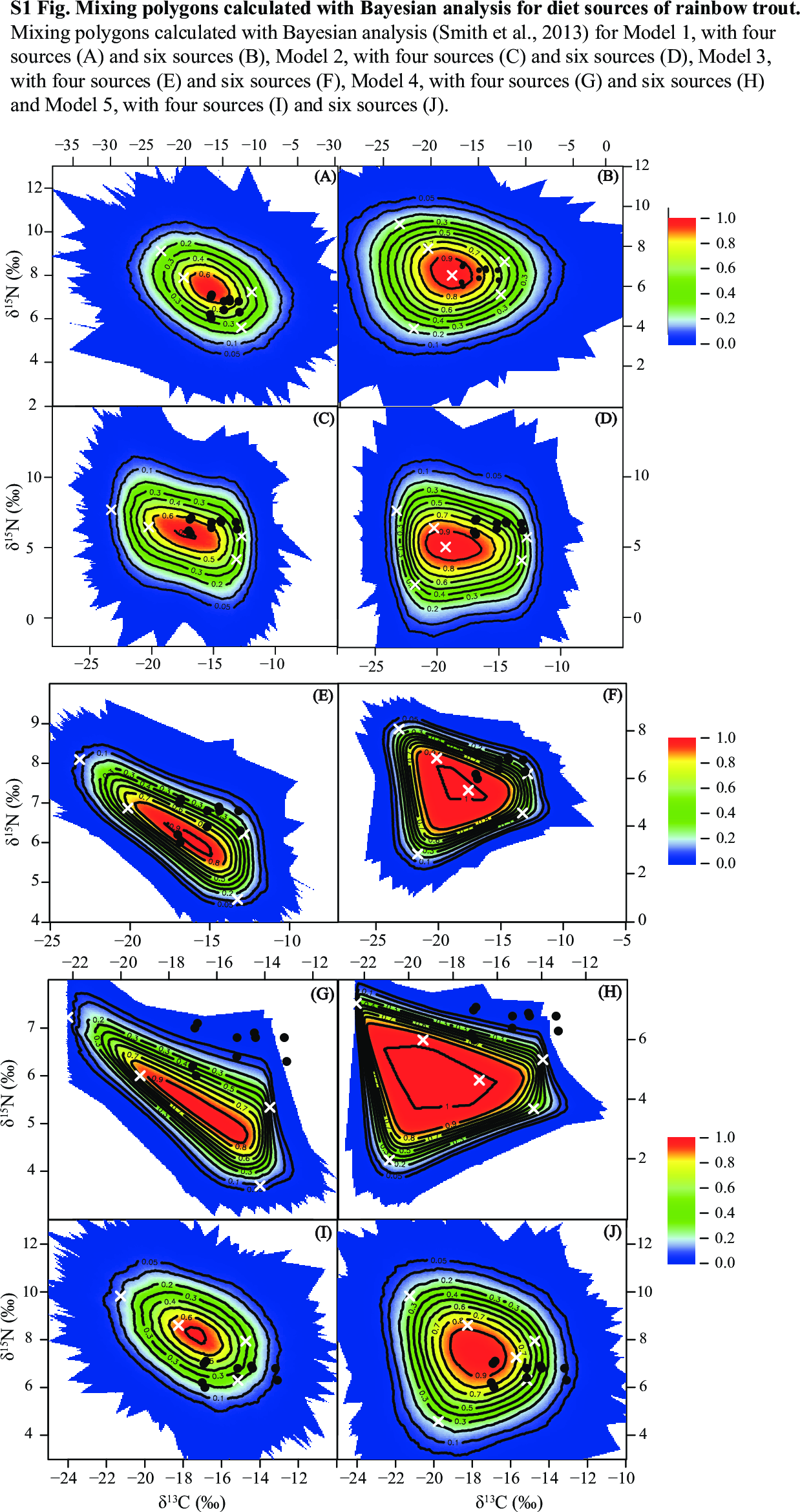

Supplement: S1 Fig — (TIFF) [file pone.0287066.s001.tiff]
