# Supplementary material for: Food web differences between two neighboring tropical high mountain lakes and the influence of introducing a new top predator
Source: PLoS One. 2023 Jun 13;18(6):e0287066. doi: 10.1371/journal.pone.0287066 (PMC10263323; doi:10.1371/journal.pone.0287066)
Supplement: S1 Table — (DOCX) [file pone.0287066.s002.docx]

**Table S1. Physicochemical, trophic, and morphometric variables of Lakes El Sol and La Luna.**

| **Variable** | **El Sol** | **La Luna** | **Reference** |
| --- | --- | --- | --- |
| Maximum depth | 9 m | 5 m | Alcocer, 2009 |
| Shoreline length | 2,363 m | 675 m) | *Alcocer et al., 2020* |
| Surface area | 19 ha | 3 ha | *Alcocer et al., 2020* |
| Relative depth | 2.77% | 5.05% | *Alcocer et al., 2020* |
| Shoreline development | 1.4 | 1.1 | *Alcocer et al., 2020* |
| Watershed area | 249 ha | 74 ha | *Alcocer et al., 2020* |
| Temperature | 10.8 ± 0.23ºC | 10.1 ± 1.6ºC | This study |
| Dissolved oxygen | 7.8 ± 0.23µgL^-1^ | 6.9 ± 0.19µgL^-1^ | This study |
| pH | (6.6 ± 0.22) | (4.7 ± 0.33) | This study |
| Electrical conductance | 47.5 ± 0.79μS cm^-1^ | 10.8 ± 0.24μS cm^-1^ | This study |
| Soluble reactive phosphorous | 14.3 ± 9.6 µg L^-1^ | 3.68 ± 4.2 µg L^-1^ | *Ibarra, 2015* |
| Dissolved inorganic nitrogen | 106.7 ± 57.7 µg L^-1^ | 30.06 ± 26.9 µg L^-1^ | *Ibarra, 2015* |
| Dissolved organic carbon | 7.6 – 8 µg L^-1^ | 1.8 – 1.9 µg L^-1^ | *Alcocer et al., 2020* |
| Chlorophyll-a | 1.5 µg L^-1^ | 0.4 µg L^-1^ | *Alcocer et al., 2020* |
