# Supplementary material for: Food web differences between two neighboring tropical high mountain lakes and the influence of introducing a new top predator
Source: PLoS One. 2023 Jun 13;18(6):e0287066. doi: 10.1371/journal.pone.0287066 (PMC10263323; doi:10.1371/journal.pone.0287066)
Supplement: S2 Table — (DOCX) [file pone.0287066.s003.docx]

**Table S2. Ecologic groups used for the food web construction of Lakes El Sol and La Luna.** († = groups with annual biomass > 5%). [phytoplankton (**P**), phytobenthos (**PB**), macroalgae (**Ma**), macrophytes (**MC**), zooplankton (**Z**) and benthic macroinvertebrates (**BMI**).

| **Group** | **Lake** | | **Ecological** | **Food source** | **Representative** |
| --- | --- | --- | --- | --- | --- |
|  | **El Sol** | **La Luna** | **characteristics** |  | **species** |
| **P1** | X^†^ | X | Small-medium size (25 - 35µm), unicellular, easy digested, medium food quality, non-motile, autotrophic | Basal resource | *Monoraphidium obtusum* |
| **P2** | X^†^ | X^†^ | Medium-large size (40-60µm), colonial, edible, medium-low food quality, non-motile, autotrophic, (extracellular mucilage production) | Basal resource | *Oocystis lacustris, Sphaerocystis schroeteri, Pseudosphaerocystis planctónica* |
| **P3** | X^†^ | X^†^ | Small size (<20µm), unicellular, flagellated, good food quality, edible, motile, mixotrophic | Basal resource | *Chlamydomonas* spp*.* |
| **P4 (A)** | X^†^ | X^†^ | Large size (>50µm), edible for big herbivorous, good food quality, autotrophic | Basal resource | *Pinnularia* spp*., Frustulia rhomboides, Stenopterobia* spp. |
| **P4 (B)** | X^†^ | X^†^ | Small size (<50µm), edible, good food quality, non-motile, autotrophic | Basal resource | *Navicula* spp*., Encyonema* spp., *Cymbella* spp*., Nitzschia* spp*.*, *Surirella* spp. |
| **P5** | X^†^ | X^†^ | Small size (<50µm), unicellular inedible, motile, mixotrophic | Basal resource | *Peridinium* spp*., Gymnodinium* sp. |
| **P6** | X^†^ | X | Large size (50 – 100µm), filamentous, inedible, non-motile, autotrophic | Basal resource | *Oedogonium* spp*., Mougeotia* sp*., Spirogyra* spp*., Zygnema* spp*.* |
| **P7** | X^†^ |  | Small-large size (20 - 60µm), unicellular, flagellated, motile, mixotrophic | Basal resource | *Euglena* spp*., Lepocinclis* spp*., Trachelomonas* spp*.* |
| **P8** |  | X^†^ | Small size, pico-plankton (<2µm), edible, non-motile, autotrophic | Basal resource | *Pico-Cyanophyta* |
| **P9** |  | X^†^ | Small size (30µm), unicellular, medium digestion, non-motile, autotrophic | Basal resource | *Cosmarium* sp*.* |
| **P10** | X^†^ | X^†^ | Small size (10 – 20µm), unicellular, flagellated, easy digestion, good food quality, motile, mixotrophic | Basal resource | *Cryptomonas* sp*., Chrysochromulina aff. parva* |
| **PB4(A)** | X | X | Medium-large size (>50µm), benthic, littoral and profundal zone | Basal resource | *Pinnularia* spp., *Frustulia* *rhomboides*, *Stenopterobia* spp. |
| **PB4(B)** | X | X | Small- medium size (<50µm), benthic, littoral and profundal zone | Basal resource | *Navicula* spp., *Encyonema* spp., *Cymbella* spp., *Nitzschia* spp |
| **MC2** | X |  | Size 8 – 12cm, submerged, littoral zone | Basal resource | *Eleocharis* sp. |
| **Ma1** | X |  | Size 0.7 – 1.5cm, aquatic, littoral zone | Basal resource | *Nitella gracillis* v. *intermedia* |
| **Ma2** | X | X | Size >100µm, aquatic, littoral zone | Basal resource | *Oedogonium* spp. *Zygnema* spp., *Spirogyra* spp., *Temnogametum iztacalense* |
| **Z1** | X^†^ | X^†^ | Size 1 - 3mm, Pelagic, Filter feeder | Bacteria, detritus, F1, F3, F10 & F8 | *Daphnia ambigua* |
| **Z2** | X^†^ | X^†^ | Size 0.1 - 0.4mm, Pelagic, Grazer and filter feeder | Bacteria, F6, F8 & F3 | *Leptodiaptomus cuauhtemoci* *Leptodiaptomu*s spp. |
| **Z3(A)** | X^†^ | X^†^ | Size 130µm, Pelagic, Filter feeder | F10, F3 & F7 | *Polyarthra* spp., *Keratella tropica* |
|  |  |  | Size 220µm, Pelagic, Filter feeder |  | *Synchaeta* spp. |
| **Z3(B)** | X | X | Size 225 - 305µm, Pelagic, Filter feeder | F10, F3, F7 & F5 | *Hexarthra bulgarica canadensis* |
| **Z4** | X | X | Size 250 - 400µm, Pelagic, Filter feeder | F5 | *Ascomorpha saltans* |
| **Z5** | X | X | Size 250 - 400µm, Pelagic, Filter feeder | F2 & F3 | *Brachionus urceolaris Brachionus bidenatus* |
| **Z6** | X | X | Size 100 - 170µm, Pelagic - benthic, Filter feeder, Filter feeder | Bacteria, detritus, F2 & F4(B) | *Lecane* spp. *Lepadella spp., Colurella spp., Bdelloidea* |
| **Z7** | X | X | Size 220 - 300µm, Benthic | F1, F2, F3, F5 & F7 | *Cephalodella* spp. |
| **Z8(A)** | X | X | Size 220 - 340µm, Benthic, Predator | Rotifers, F1, F2, F3, F7 & F4(A) | *Notommata* spp. |
| **Z8(B)** | X | X | Size 100 - 200µm, benthic littoral zone, Predator | Rotifers, F6, F4(A) | *Trichocerca* spp. |
| **BMI1(A)** | X^†^ |  | 1 - 7 cm, Collector-gatherer | Detritus, bacteria, F4(A), F4(B), & F2 | *Lumbriculus variegatus* |
| **BMI1(B)** | X^†^ | X^†^ | 0.5 – 5cm, Collector-gatherer | Detritus, bacteria, F4(A), F4(B) & F2 | *Limnodrilus hoffmeisteri* |
| **BMI2** | X^†^ |  | 2 mm, Collector-gatherer | Detritus & bacteria | *Tubifex tubifex* |
| **BI3** | X^†^ |  | 0.2 – 1.3mm, Collector-gatherer | Detritus, bacteria, F4(A) & F4(B) | *Nais pardalis* |
| **BMI4** | X^†^ | X^†^ | 0.8 – 1mm, Collector- filter feeder | Detritus, pollen, F1, F2, F3, F9, F4(A) & F4/B) | *Tanytarsus* sp. |
|  |  |  |  | Detritus, F4(A) & F4(B) | *Pagastia* sp. |
| **BMI5** | X^†^ |  | 2.2 - 4.2mm, Collector- filter feeder, (deposit feeder) | Detritus, F2, F3, F9, & F4(B) | *Pisidium casertanum* |
| **BMI6** | X^†^ |  | 1.8 - 3mm, Collector- filter feeder, (deposit feeder) | Detritus, bacteria, macrophytes, F2, & F6 | *Herpetociprys* sp. |
| **BMI7** | X^†^ |  | 0.3 – 3mm, Predator | F4(B), F4(A), F6, 0.3 – 3mm | *Hydra vulgaris* |
| **Herbivores - detritivores, size <500 µm** | | | | | |
| **BMI8(A)** | X^†^ |  | <500µm, Predator- detritivore | F6, unicellular algae, detritus, bacteria | *Monhystera* spp., *Eumonhystera dispar, Plectus* spp. |
| **BI8(B)** | X^†^ | X^†^ | <500µm, Shredder | Detritus, bacteria, F6, unicellular algae, F4(A) & F4(B) | *Prismatolaimus intermedius*, *Aphanolaimus aquaticus, Tobrilus sp.* |
| **Predators, size <500µm** | | | | | |
| **BMI9** | X |  | <500µm, Omnivore | Algae, plants, rotifers & BMI | *Tylenchus* spp., *Laimydorus pseudostagnalis* |
| **BMI10** | X^†^ | X^†^ | <500µm, Predator | IB9, IB10, rotifers | *Ironus* spp. |
| **BMI11** | X |  | 6 – 10mm, Scrapers | Periphyton, macrophytes & leaf litter | *Physa* sp. |
