# Supplementary material for: Food web differences between two neighboring tropical high mountain lakes and the influence of introducing a new top predator
Source: PLoS One. 2023 Jun 13;18(6):e0287066. doi: 10.1371/journal.pone.0287066 (PMC10263323; doi:10.1371/journal.pone.0287066)
Supplement: S3 Table — (DOCX) [file pone.0287066.s004.docx]

**Table S3. The minimal data set of stable isotope data (δ^13^C and δ^15^N) for the major Lakes El Sol and La Luna groups.** Dap = *Daphnia ambigua*, Gas = *Phys* sp., Lep = *Leptodiaptomus cuauhtemoci*, Malg = macroalgae (Oedogoniaceae and Zygnemataceae), MphyC_3_ = macrophytes with C_3_ metabolism, MphyC_4_ = macrophytes with C_4_ metabolism, Phyt = phytoplankton, SOM = sediment organic matter (L= littoral and P= profundal), OliH = *Limnodrilus hoffmeisteri*, OliT = *Tubifex tubifex*, OliV = *Lumbriculus variegatus*, Tro = *Oncorhynchus mikyss*, Zoo = bulk zooplankton.

| **Lake** | **Group/**  **species** | **δ^13^C** | **δ^15^N** | **Lake** | **Group/**  **species** | **δ^13^C** | **δ^15^N** |
| --- | --- | --- | --- | --- | --- | --- | --- |
| El Sol | Tro | -17.92 | 7.3 | El Sol | Malg | -10.06 | -1.6 |
| El Sol | Tro | -17.33 | 7.5 | El Sol | Malg | -7.88 | ND |
| El Sol | Tro | -17.21 | 7.3 | El Sol | MphyC_3_ | -22.6 | -2.6 |
| El Sol | Tro | -16.87 | 6 | El Sol | MphyC_3_ | -26.1 | -0.5 |
| El Sol | Tro | -17.01 | 6.2 | El Sol | MphyC_3_ | -26.1 | -1.2 |
| El Sol | Tro | -16.81 | 7.1 | El Sol | MphyC_3_ | -25.3 | -2.2 |
| El Sol | Tro | -16.92 | 7 | El Sol | MphyC_3_ | -26.23 | -1.6 |
| El Sol | Tro | -15.17 | 6.4 | El Sol | MphyC_3_ | -26.42 | 1.5 |
| El Sol | Tro | -15.16 | 6.8 | El Sol | MphyC_4_ | -11.17 | -1.2 |
| El Sol | Tro | -14.44 | 6.9 | El Sol | Phyto | -17.29 | 0 |
| El Sol | Tro | -14.39 | 6.8 | El Sol | Phyto | -14.81 | 0.7 |
| El Sol | Tro | -13.09 | 6.3 | El Sol | Phyto | -17.19 | 0.8 |
| El Sol | Tro | -13.19 | 6.8 | El Sol | Phyto | -17.25 | 2.48 |
| El Sol | OliiV | -14.02 | 1.2 | El Sol | Phyto | -18.81 | 0.51 |
| El Sol | OliiV | -17.78 | 1.08 | El Sol | Phyto | -24.16 | 1.69 |
| El Sol | OliiV | -14.03 | 1.04 | El Sol | SOMP | -25.54 | -0.6 |
| El Sol | OliiV | -11.66 | 1.3 | El Sol | SOMP | -20.38 | -1.6 |
| El Sol | OliiV | -11.55 | 0.8 | El Sol | SOMP | -20.63 | -1.4 |
| El Sol | OliiV | -12.49 | 1 | El Sol | SOMP | -20.56 | -1.3 |
| El Sol | OliiV | -11.73 | 1.2 | El Sol | SOMP | -20.65 | -1.2 |
| El Sol | OliiV | -11.59 | 1.2 | El Sol | SOML | -7.35 | -0.9 |
| El Sol | OliiV | -11.76 | 1.5 | La Luna | Dap | -28.55 | 3.98 |
| El Sol | OliiT | -18.84 | 2.26 | La Luna | Dap | -29.12 | 5.3 |
| El Sol | OliiT | -18.9 | 3 | La Luna | Dap | -27.93 | 2.8 |
| El Sol | OliiT | -17.78 | 1.08 | La Luna | Dap | -26.98 | 2.7 |
| El Sol | OliiH | -22.02 | -0.95 | La Luna | OliiV | -21.58 | -0.1 |
| El Sol | OliiH | -22.09 | -1.07 | La Luna | OliiV | -21.53 | -0.4 |
| El Sol | OliiH | -23.14 | 0 | La Luna | OliiH | -22.87 | 0.7 |
| El Sol | OliiH | -23.02 | -0.25 | La Luna | OliiH | -22.48 | 0.8 |
| El Sol | Gas | -11.84 | 2.4 | La Luna | Malg | -16.02 | -6.70 |
| El Sol | Gas | -12.04 | 3.2 | La Luna | Malg | -15.81 | -7.2 |
| El Sol | Dap | -21.12 | 3.32 | La Luna | Malg | -17.61 | -7.9 |
| El Sol | Dap | -20.98 | 3.6 | La Luna | Malg | -17.72 | -8.1 |
| El Sol | Lep | -24.09 | 4.37 | La Luna | SOMP | -24.46 | -0.7 |
| El Sol | Lep | -24.04 | 5 | La Luna | SOMP | -24.52 | -0.6 |
| El Sol | Zoo | -22.8 | 5.1 | La Luna | SOMP | -24.88 | -0.7 |
| El Sol | Zoo | -23.63 | 6.7 | La Luna | SOMP | -24.7 | -0.9 |
| El Sol | Zoo | -23.72 | 5.54 | La Luna | Phyto | -21.06 | 1.36 |
| El Sol | Zoo | -23.11 | 4.45 | La Luna | Phyto | -17.42 | 1.54 |
| El Sol | Malg | -10.41 | -1.3 | La Luna | Phyto | -21.16 | 2.97 |
| El Sol | Malg | -10.7 | -1.8 | La Luna | Phyto | -20.50 | 1.99 |
| El Sol | Malg | -10.1 | -1.5 |  |  |  |  |
